# Molecular insights into the interaction mechanism of endocrine-disrupting chemicals and DNA in laccase-induced polymerization transfer

**DOI:** 10.1093/pnasnexus/pgaf148

**Published:** 2025-05-12

**Authors:** Kai Sun, Zeyu Shi, Lingzhi Dai, Youbin Si, Junchao Ma, Hui Lin, Han-Qing Yu

**Affiliations:** College of Resources and Environment, Anhui Agricultural University, Hefei 230036, China; CAS Key Laboratory of Urban Pollutant Conversion, Department of Environmental Science and Engineering, University of Science and Technology of China, Hefei 230026, China; College of Resources and Environment, Anhui Agricultural University, Hefei 230036, China; College of Resources and Environment, Anhui Agricultural University, Hefei 230036, China; College of Resources and Environment, Anhui Agricultural University, Hefei 230036, China; College of Resources and Environmental Sciences, Nanjing Agricultural University, Nanjing 210095, China; Research Center for Eco-Environmental Engineering, Dongguan University of Technology, Dongguan 523808, China; CAS Key Laboratory of Urban Pollutant Conversion, Department of Environmental Science and Engineering, University of Science and Technology of China, Hefei 230026, China

**Keywords:** DNA, endocrine-disrupting chemicals, laccase polymerization, interaction mechanism, genotoxicity

## Abstract

Endocrine-disrupting chemicals (EDCs), such as 17β-estradiol (E2) and bisphenol A (BPA), can induce DNA damage, leading to genomic instability and cell death. Laccase, an enzyme secreted by diverse organisms, plays a critical role in mitigating the cytotoxicity of these contaminants. Despite its importance, the dynamic evolution and interaction mechanisms of EDCs and DNA in laccase catalysis remain poorly understood. This study investigates the interactions between EDCs and DNA during laccase-induced polymerization transfer at a molecular level. As the DNA concentration was increased from 0 to 7.575 nM, the pseudo-first-order kinetic constants for E2 and BPA decreased by 2.03 and 2.10 times, respectively. DNA-bound EDCs disrupted the catalytic activity and stability of laccase, thereby delaying the polymerization transfer rate of EDCs. E2 and BPA bound to DNA base pairs via groove and intercalative modes, respectively. Laccase-induced polymerization reduced damage to the DNA helix and base stacking caused by EDC binding. Moreover, the resulting DNA–EDC-precipitated polymers, formed through continuous laccase polymerization, exhibited denser and more complex structures compared with spherical EDC-precipitated polymers, confirming DNA encapsulation and/or binding. This work underscores the intramolecular mechanisms of EDC interaction with DNA in vitro during the laccase-induced polymerization, offering efficient ways to mitigate the genotoxicity of EDCs.

**Significance Statement**

Laccase-induced polymerization transfer is rifely applied for decontaminating and detoxifying endocrine-disrupting chemicals (EDCs). Still, the dynamic processes and intermolecular interactions of EDCs and DNA in laccase polymerization remain largely unexplored. Herein, the interactions between EDCs and DNA during laccase-induced polymerization transfer are unmasked at the molecular level. DNA observably alters the kinetics and mechanisms of laccase-induced EDC polymerization through the specified binding interactions, leading to the structural changes in DNA and EDC polymers. By forming low-toxic and ultimately nontoxic EDC-precipitated polymers, the DNA damage and mutation triggered by EDCs is mitigated. This work elucidates the molecular interactions between EDCs and DNA in vitro during the laccase-induced polymerization, suggesting laccase's potential role in mitigating the genotoxic effects of EDCs in vivo.

## Introduction

Endocrine-disrupting chemicals (EDCs) are often described as “a ticking time bomb threatening human survival” (1). Globally, the widespread presence of both natural and synthetic EDCs, such as 17β-estradiol (E2) and bisphenol A (BPA), has raised significant concerns about their detrimental effects on wildlife and human health (2, 3). Due to their persistent and bioaccumulative nature, EDCs represent a latent crisis for both environmental and human health. Moreover, these chemicals undergo transformations in the environment to form secondary pollutants, which are often more toxic than their precursors (4). These compounds, entering organisms directly or indirectly, have been linked to reproductive disorders, deformities in sexual organs, sex reversal, and feminization in wildlife species (5). Recent studies also correlate the exposure to EDCs with increasing rates of large-bowel, breast, cervical, and ovarian cancers in women and declining sperm count and motility in men (6, 7).

The hazardous effects of EDCs are attributed to their interactions with DNA, disrupting DNA repair, replication, and transcription processes, and thereby causing enhanced DNA damage and cell death (8, 9). Understanding the dynamic processes and intermolecular interactions of EDCs with DNA is crucial for elucidating their mechanisms of genotoxicity.

DNA, the carrier and transmitter of hereditary information in nearly all living systems, plays a central role in vital biogenetic processes, such as gene expression, transcription, mutation, and carcinogenesis (10). Composed of pentose sugar, phosphoric acid, and nitrogenous bases, DNA's genetic information is encoded by specific sequences of these bases. The stability of the DNA helix is significantly maintained by the stacking and hydrogen-bonding interactions between nucleobases (11). Thus, the interaction of EDCs with DNA could directly impact its structure and function, leading to DNA damage in exposed cells and altering its existing functions (1). Organic pollutants of biological importance are known to interact with DNA through noncovalent interactions, including electrostatic interactions, groove binding, and intercalative binding (12–14).

Laccase (EC 1.10.3.2, oxygen oxidoreductase), a member of the multicopper oxidase superfamily, is naturally secreted by a variety of organisms such as bacteria, fungi, plants, and insects. It has garnered attention for its high activity, low energy requirements, and nontoxicity in degrading harmful phenolic substances (15, 16). Laccase catalyzes the oxidation of EDCs, facilitating the formation of phenoxy radicals, which subsequently polymerize into dimers, trimers, tetramers, oligomers, and polymers (17, 18). Despite its potential, the detailed understanding of the dynamic evolution and molecular mechanisms of interaction between EDCs and DNA during laccase-induced polymerization remains scant.

This work aims to elucidate, at the molecular level, the dynamic processes and interaction mechanisms between EDCs and DNA during the laccase-induced polymerization transfer. We explored the reaction kinetics of EDCs under varying DNA concentrations using a pseudo-first-order kinetic model and utilized high-resolution mass spectrometry (HRMS) to identify key oxidation products. Advanced spectroscopic techniques, such as UV-Vis spectroscopy, attenuated total reflectance-Fourier transform infrared spectroscopy (ATR-FTIR), and circular dichroism (CD) spectroscopy, were employed to reveal the dynamic interactions of DNA with EDCs during polymerization. Atomic force microscopy (AFM) was used to unmask the dynamic variations in DNA morphology on interaction with EDCs and their oligomers from the molecular perspective. Further, the precipitated polymers were characterized using scanning electron microscopy (SEM) equipped with an energy-dispersive X-ray spectrometer (EDS), FTIR, and ^13^C-nuclear magnetic resonance spectroscopy (^13^C-NMR). Molecular dynamics simulation (MDS) was conducted to verify the intermolecular interactions between DNA and the oligomers of EDCs. The cytotoxicity of DNA–EDC solutions treated by laccase-induced polymerization was evaluated over time. By uncovering the dynamic evolution and intermolecular interactions of EDCs and DNA in vitro during the laccase-induced polymerization, this study offers insights into molecular strategies for mitigating the genotoxicity of EDCs.

## Results and Discussion

### Reaction kinetics and polymerization transfer of EDCs induced by laccase in the presence of DNA

**Fig. 1.**
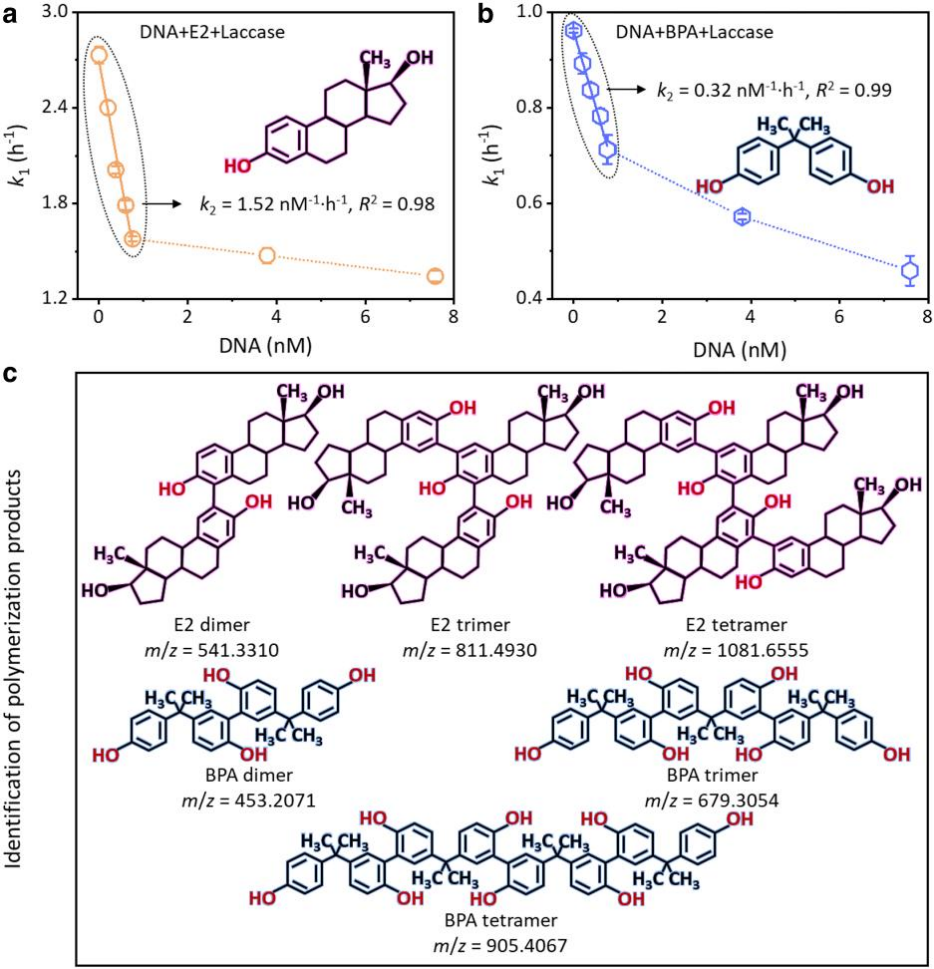
Impact of DNA on oxidation kinetics and oligomerization of EDCs by laccase. a) Graph depicting the relationship between the pseudo-first-order rate constant (*k*_1_) for E2 and varying concentrations of DNA (0–0.7575 nM). b) Graph showing the relationship between the pseudo-first-order rate constant (*k*_1_) for BPA and varying concentrations of DNA (0–0.7575 nM). c) Chart of key oligomerization products identified from the oxidation of EDCs during laccase catalysis. Error bars represent SDs, with *n* = 3 replicates. The dotted ovals indicate the fitting parameters of the pseudo-second-order kinetics (Fig. 1a and b).

Increasing DNA concentrations significantly impeded the laccase-induced single-electron oxidation transfer of EDCs. Figure S1 illustrates that the oxidation rates of E2 and BPA decreased from 97.25 to 80.37% and 71.64 to 54.18%, respectively, after a 3-h reaction, as the DNA concentration was increased from 0 to 7.575 nM. The laccase-mediated oxidation of EDCs adhered to a pseudo-first-order kinetics model within 1 h, with *k*_1_ values for E2 and BPA decreasing by factors of 2.03 and 2.10, respectively, as the DNA concentration was increased (*R*^2^ = 0.96–0.99). This suggests that laccase activity was inversely related to the DNA concentration during the enzyme-catalyzed oxidation of EDCs (Fig. S2). Linear regression analysis further elucidated the dependency of the *k*_1_ value on low DNA concentration (0–0.7575 nM), revealing a linear relationship (*R*^2^ ≥ 0.98) and calculated pseudo-second-order rate coefficients (*k*_2_) for E2 and BPA of 1.52 and 0.33 nM^−1^·h^−1^, respectively (Fig. 1a and b).

High levels of phenoxy radicals, produced by the laccase-induced oxidation of EDCs, facilitated the polymerization transfer. HRMS identified the polymerization products, revealing their molecular architectures through computational analysis (Table S1 and Fig. 1c). Notably, three major E2 oligomers—dimer (*m*/*z* = 541.3310), trimer (*m*/*z* = 811.4930), and tetramer (*m*/*z* = 1,081.6555)—suggested the pattern of *n* × *M* − 2(*n* − 1) for EDC polymerization products, where *n* is the number of polymerized EDC monomers and *M* is the molecular mass of the monomer. Additionally, a peak at *m*/*z* = 269.1548 indicated the formation of estrone (E1) by deprotonation, accounting for <2.4% of the initial E2 after 1 h of reaction. This pattern was consistent with the laccase-induced polymerization of BPA, suggesting that polymerization transfer is the predominant pathway in EDC oxidation by laccase, regardless of the presence of DNA.

### Interaction of DNA with EDCs in laccase-induced polymerization transfer

**Fig. 2.**
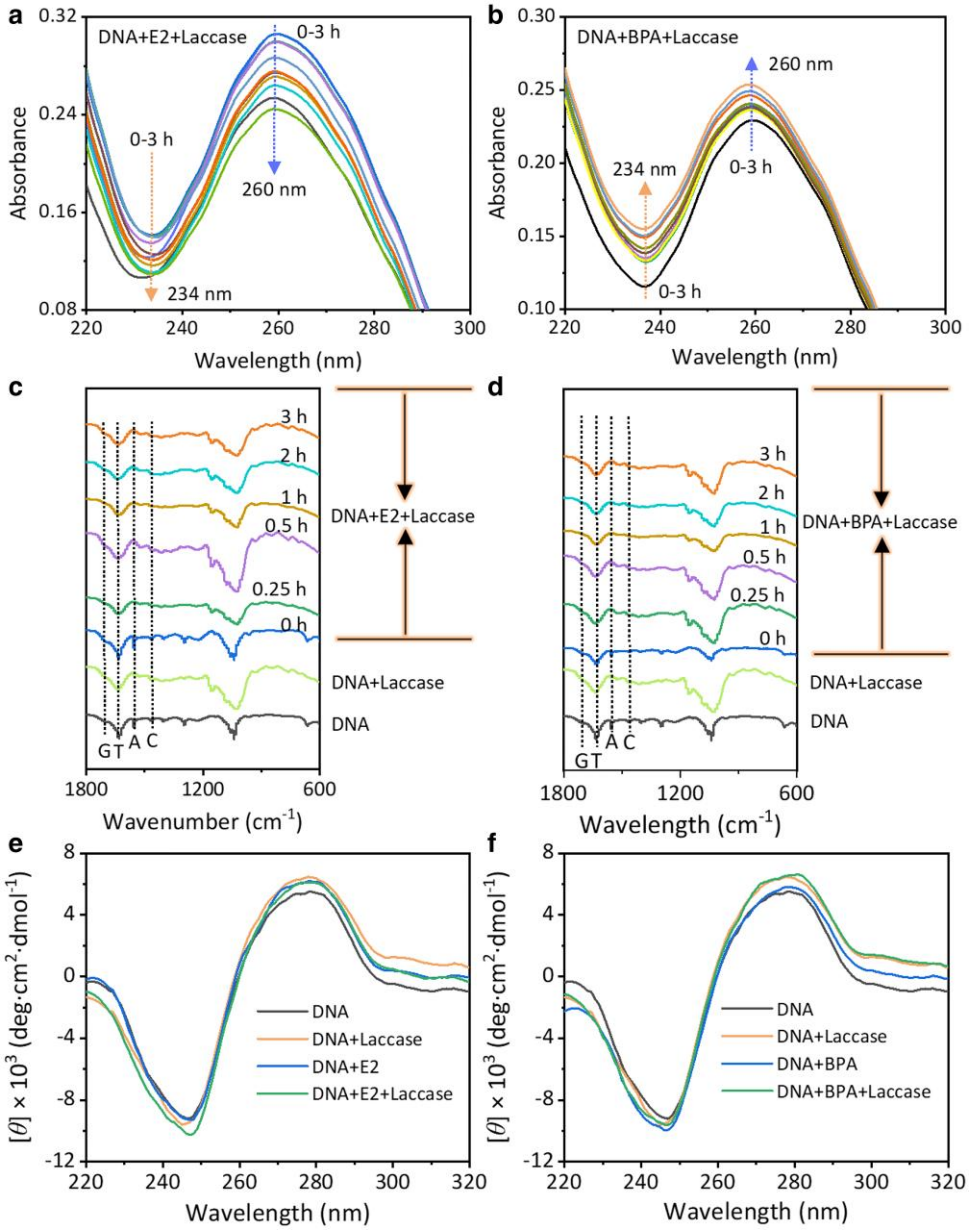
Intermolecular interactions and conformational changes of DNA in laccase-induced polymerization of EDCs. a) UV-Vis spectra demonstrating the impact of laccase-induced polymerization of E2 on DNA structure. b) UV-Vis spectra showing the influence of laccase-induced polymerization of BPA on DNA structure. c) Time-dependent FTIR spectra illustrating DNA binding with E2 and its oligomers during polymerization. d) Time-dependent FTIR spectra showing DNA binding with BPA and its oligomers during polymerization. e) CD spectra of DNA during E2 polymerization, highlighting changes in DNA conformation. f) CD spectra of DNA during BPA polymerization, depicting structural alterations. The dotted arrows indicate decreasing and increasing trends over time, respectively (a and b). The dotted lines indicate the spectral changes of guanine (G), thymine (T), adenine (A), and cytosine (C) bases in DNA at 1,691, 1,648, 1,605, and 1,485 cm^−1^, respectively (c and d).

UV-Vis absorption spectroscopy confirmed the interaction modes between DNA and EDCs. The UV-Vis absorption spectra of DNA displayed two peaks at 234 and 260 nm (Fig. S3). The addition of E2 resulted in a hyperchromic effect at 260 nm, indicating damage to the DNA helix through a nonintercalative interaction mode. The degree of E2 polymerization transfer correlated negatively with the intensities of the characteristic DNA peaks (Fig. 2a). In contrast, the interaction with BPA showed a hypochromic effect, suggesting an intercalative binding that involves strong π–π aromatic stacking interactions between BPA and the phosphate backbone of the DNA helix (19). The degree of BPA polymerization transfer correlated positively with DNA peak intensities (Fig. 2b). Clearly, laccase-induced polymerization effectively reduced the concentration of EDCs and mitigated their interaction with DNA.

The molecular fingerprint of DNA is located in the spectral region of 1,800–700 cm^−1^ due to nitrogenous bases, phosphate, and deoxyribose stretching vibrations. Binding of EDCs to DNA bases was investigated by analyzing spectral changes at 1,691, 1,648, 1,605, and 1,485 cm^−1^ that were attributed to guanine, thymine, adenine, and cytosine base vibrations, respectively. FTIR spectra explored the binding sites of DNA and EDCs, with characteristic DNA peaks indicating interactions at thymine and adenine bases, which appeared to be primary binding sites for EDCs (Fig. 2c and d). CD spectroscopy further revealed conformational changes in DNA induced by EDCs, highlighting the distinct interaction modes of E2 and BPA with DNA. The upward shift in the positive peak suggested enhanced base stacking due to groove binding by E2, while the downward shift in the negative peak for BPA indicated intercalative binding (Fig. 2e and f).

**Fig. 3.**
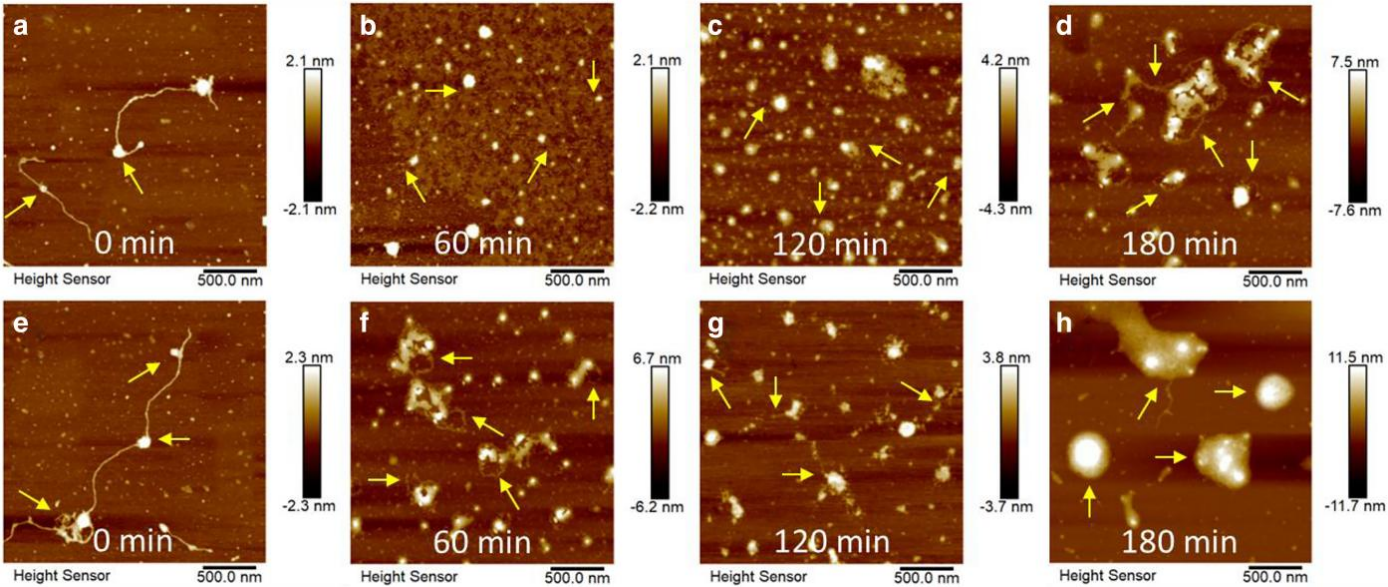
Binding mechanisms of EDCs and their oligomers to DNA observed by AFM, implying DNA encapsulation and/or binding during polymerization. a–d) AFM topographical images of DNA binding with E2 and its oligomers at different polymerization times (a = 0 min, b = 60 min, c = 120 min, and d = 180 min). e–h) AFM topographical images of DNA binding with BPA at different polymerization times (e = 0 min, f = 60 min, g = 120 min, and h = 180 min). The arrows indicate representative intramolecular interactions of DNA with EDCs and their oligomers.

AFM does not require any additional sample pretreatment to observe samples in liquids, making it exceptionally suitable for DNA imaging and therefore plays an important role in probing the intermolecular interactions of EDCs and DNA during the laccase-induced polymerization. AFM offered direct evidence of DNA binding with EDCs at the molecular level (Fig. 3). No obvious change in the contour length of DNA was observed in the presence of E2, confirming that E2 bound to DNA by groove mode (Fig. 3a). However, the interaction of intercalating BPA with DNA could lead to an increase in the contour length of DNA by bridging two or more DNA molecules (Fig. 3e). Further, the enhanced degree of EDC polymerization boosted DNA encapsulation and/or binding, forming larger spherical particles (Fig. 3b–d and f–h).

### Architectural characteristics of DNA–EDC-precipitated polymers

**Fig. 4.**
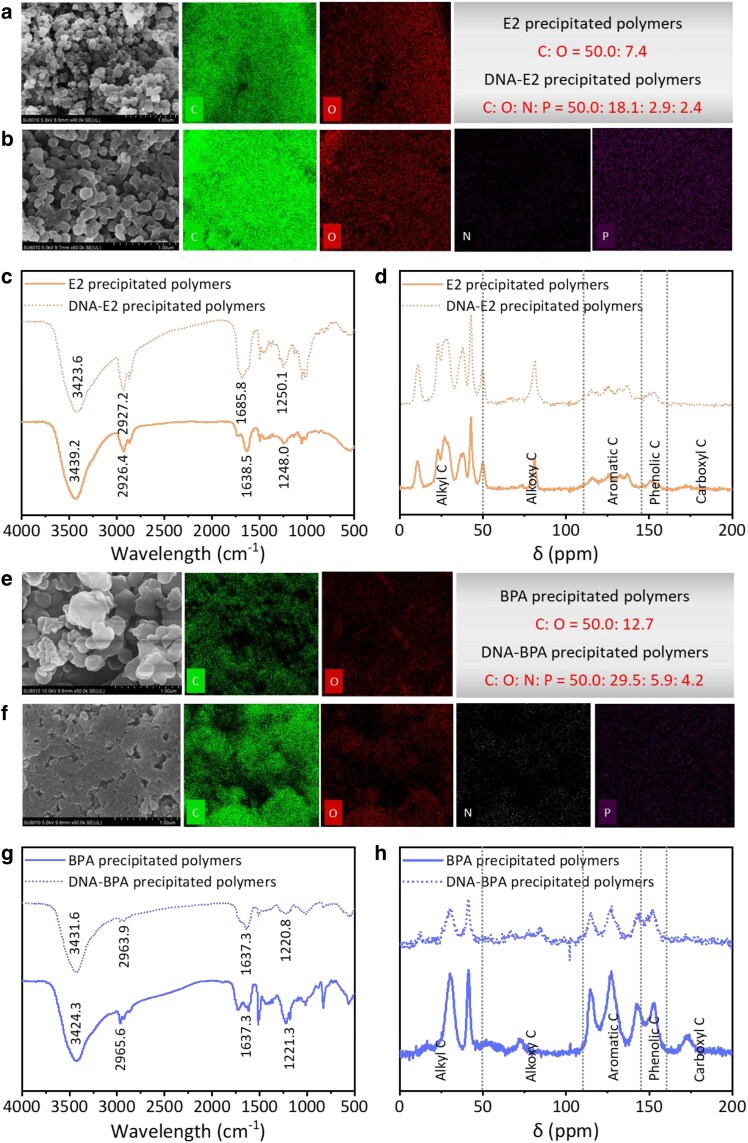
Architectural characteristics of DNA–EDC-precipitated polymers. a) SEM images and elemental EDX mapping of E2-precipitated polymers. b) SEM images and elemental EDX mapping of DNA–E2-precipitated polymers, illustrating more complex structures. c) FTIR spectra of DNA–E2-precipitated polymers, showing distinct vibrational signatures. d) ^13^C-NMR spectra of DNA–E2-precipitated polymers, identifying carbon environments. e) SEM images and elemental EDX mapping of BPA-precipitated polymers. f) SEM images and elemental EDX mapping of DNA–BPA-precipitated polymers, displaying enhanced aggregation. g) FTIR spectra of DNA–BPA-precipitated polymers, indicating functional group interactions. h) ^13^C-NMR spectra of DNA–BPA-precipitated polymers, highlighting chemical shifts and structural complexity. The dotted lines indicate the characteristic peaks of alkyl carbon, alkoxy carbon, aromatic carbon, phenolic carbon, and carboxyl carbon in the ranges of 0–50, 50–110, 110–145, 145–160, and 160–200 ppm, respectively (d and h).

SEM analysis showed that compared with the spherical E2-precipitated polymers, DNA–E2-precipitated polymers displayed a denser and thicker morphology, indicative of tighter and more complex structural integration (Fig. 4a and b). EDS confirmed a lower carbon-to-oxygen ratio in the DNA–E2-precipitated polymers compared with those without DNA, suggesting more extensive oxygen bonding. A similar phenomenon was also found in the BPA and DNA–BPA-precipitated polymers (Fig. 4e and f). As demonstrated in Fig. 4c and g, the EDC-precipitated polymers displayed the functional characteristics of the aromatic skeleton, phenolic –OH, and ether linkage. By comparison, the intensities and positions of the above functional groups in the DNA–EDC-precipitated polymers changed unmistakably. ^13^C-NMR spectra of the precipitated polymers revealed a predominance of aromatic and phenolic carbon, indicating that DNA was potentially encapsulated and/or bound to the EDC-precipitated polymers during the laccase-induced polymerization and thus facilitated more complex carbon backbone formations (Fig. 4d and h).

The dynamics of polymerization and the molecular interactions were extensively analyzed to elucidate the complex behavior of EDCs in the presence of DNA during laccase catalysis. These insights provide a deeper understanding of the intramolecular mechanisms through which laccase-mediated processes may mitigate the genotoxicity of EDCs, highlighting the potential applications of laccase polymerization for the decontamination and detoxification of EDCs at the molecular level.

### Interaction of DNA with EDCs and their oligomers

**Fig. 5.**
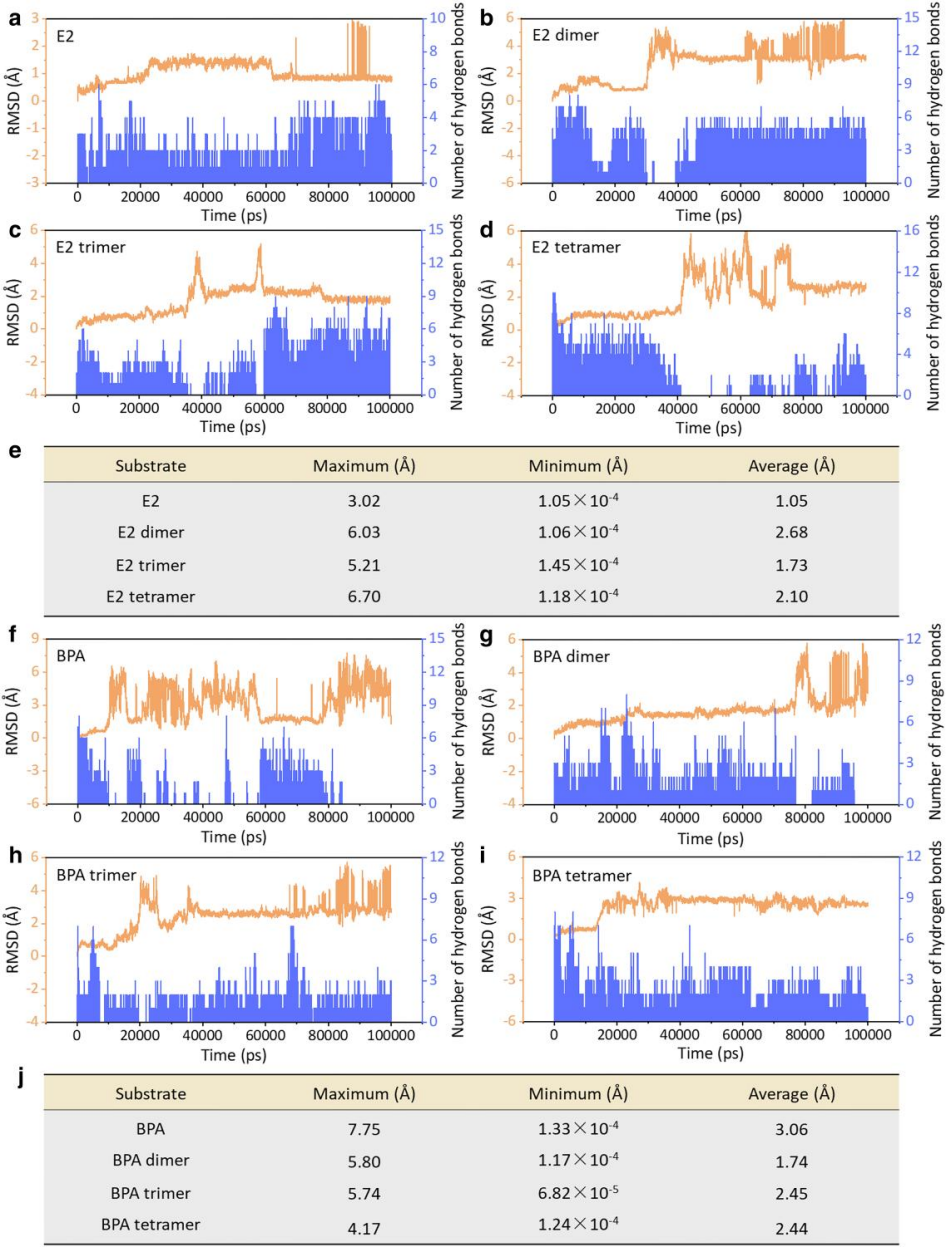
Rmsd and hydrogen bond analysis for DNA-bound EDCs and their oligomers. a–d) Rmsd values and hydrogen bond counts for DNA-bound E2, E2 dimer, E2 trimer, and E2 tetramer systems, respectively. e) Average rmsd values comparing DNA-bound E2 and its oligomeric forms. f–i) Rmsd values and hydrogen bond counts for DNA-bound BPA, BPA dimer, BPA trimer, and BPA tetramer systems, respectively. j) Average rmsd values for DNA-bound BPA and its oligomers, highlighting stability differences.

To support the above experimental results, we further explored the stability and position changes of EDCs and their oligomers binding to DNA by computer-aided MDS. Root-mean-square deviation (rmsd) serves as a measure of the stability and interaction between DNA and substrates. Higher rmsd values suggest more unstable interactions. In this study, the interactions of DNA with EDCs and their oligomers (dimer, trimer, and tetramer) were examined over a 100-ns MDS period. As illustrated in Fig. 5a–e, rmsd values for DNA-bound E2 and its oligomers ranged from 0 to 7.0 Å. The average rmsd for DNA-bound E2 was 1.05 Å, indicating more stable interactions compared with DNA-bound E2 dimer (2.68 Å), trimer (1.73 Å), and tetramer (2.10 Å). Hydrogen bonds, crucial for the affinity and selectivity of the DNA-substrate complex, diminished in number from the monomer to the oligomers, underscoring the enhanced stability of DNA-bound E2. Conversely, for BPA, the mean rmsd values increased with oligomerization; DNA-bound BPA monomer exhibited an rmsd of 3.06 Å, greater than its oligomers—dimer (1.74 Å), trimer (2.45 Å), and tetramer (2.44 Å; Fig. 5f–j), correlating with an increase in hydrogen bonds with higher molecular weights, indicating greater stability in DNA-bound BPA oligomers.

**Fig. 6.**
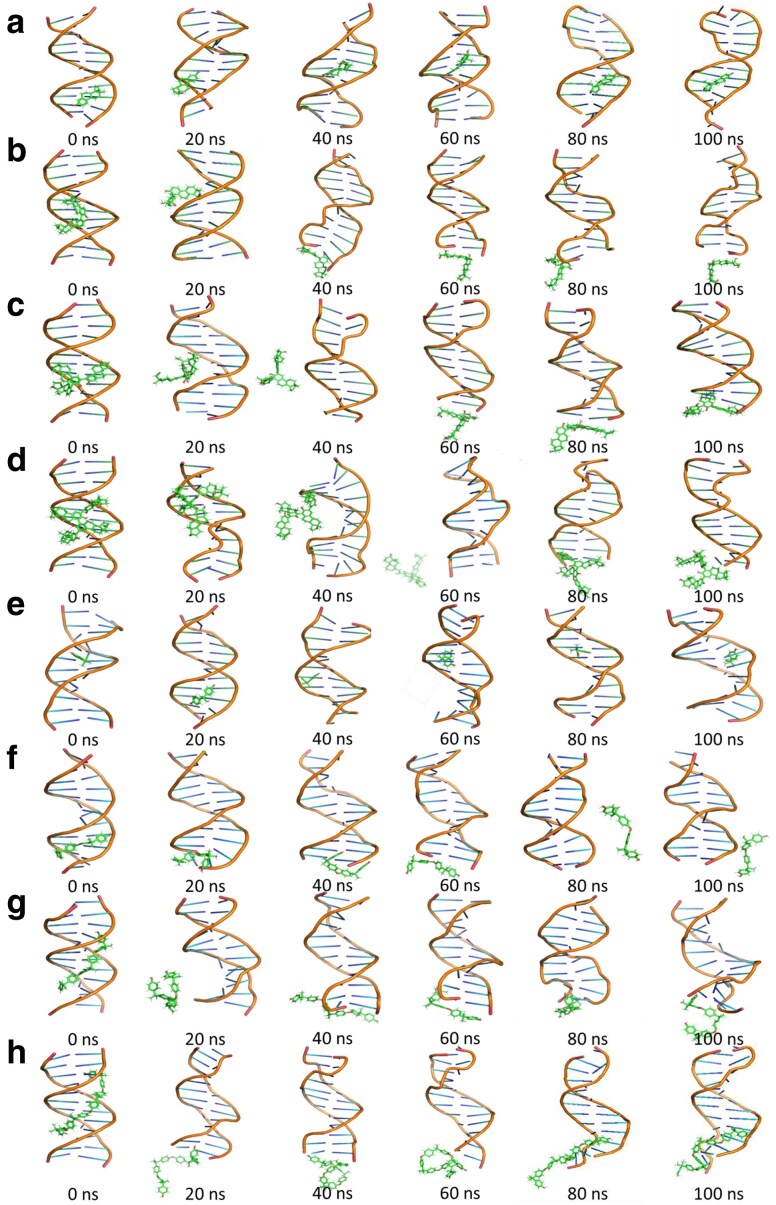
Position variations of EDCs and their oligomers relative to DNA over simulation time, confirming that the binding of EDCs and their dimers, trimers, and tetramers to the DNA was unstable during the simulation progress. a–d) The specialized binding sites of E2, E2 dimer, E2 trimer, and E2 tetramer at 0–100 ns. e–h) The specialized binding positions of BPA, BPA dimer, BPA trimer, and BPA tetramer at 0–100 ns.

As mentioned above, hydroxyl substitution facilitated the binding of EDCs and their oligomers to DNA, but the intermolecular binding was relatively unstable (rmsd > 1). Figure 6 shows the position changes of EDCs and their dimers, trimers, and tetramers relative to DNA at different simulation times. As the MDS time was increased, EDCs and their oligomers gradually deviated from the initial binding sites of DNA. Furthermore, these results also disclosed abnormal DNA structures, which were consistent with the CD spectroscopy. Overall, the MDS results confirm that EDCs and their oligomers unstably bound to their corresponding binding sites during the simulation process.

### Implications for environmental applications

The dynamic interactions and mechanisms of DNA with EDCs during the laccase-induced polymerization remain poorly understood. Laccase facilitates the one-electron oxidation of EDCs, predominantly leading to polymerization products, while DNA hampers this transfer by interacting with functional groups of DNA molecules, such as base pairs and phosphate skeletons. This interaction forms complexes of DNA-bound EDCs, which obstruct laccase's catalytic activity and stability, thereby slowing the polymerization rate of EDCs. In contrast, the polymerization products of EDCs, also bound to DNA via hydrogen bonds, form denser and thicker structures compared with those without DNA, suggesting potential encapsulation and/or binding within the DNA–EDC polymers. Understanding these dynamic processes and interactions provides insights into mitigating the genotoxicity and cancer risks associated with EDCs.

The EDC polymers precipitated through laccase-induced polymerization play a crucial role in reducing the cytotoxic effects of EDCs (20). The phytotoxicity assessments of DNA–EDC solutions treated through laccase-induced polymerization, illustrated in Fig. S4, demonstrate significant reductions in EDC-induced inhibitory effects on seed germination and root elongation. For instance, the germination index (GI) of wheat seeds treated with E2 increased from 12.85% (severe inhibition) to 87.48% (no inhibition) over a 9-h laccase polymerization period. The addition of DNA maintained a GI of 57.69%, even after 9 h. Furthermore, the cytotoxicity of polymerization products diminished over time, showcasing the potential of laccase-induced polymerization in alleviating the genotoxic effects of EDCs. Significantly, microbial enzymes may degrade DNA, resulting in the release of DNA-bound EDCs. Future studies are required to explore the stability of DNA-bound EDCs and their oligomers, as well as the long-term effects of the complexes in the environment. Furthermore, the in vivo interactions of DNA with EDCs should also be explored to uncover the self-detoxification mechanisms of laccase-producing organisms at a genomic level.

## Conclusions

In summary, this work provides comprehensive insights into the complex interactions between EDCs and DNA in the context of laccase-mediated reactions. The findings reveal that DNA significantly alters the kinetics and mechanisms of EDC polymerization, potentially offering a novel approach to mitigate the environmental impact and genotoxicity of these harmful compounds. By elucidating the specific binding interactions and the resulting structural changes in DNA and EDC polymers, this research contributes to a deeper understanding of the intramolecular mechanisms of EDC interaction with DNA in vitro during the laccase-induced polymerization and opens new avenues for the development of more effective decontamination and detoxification strategies in vivo.

## Materials and methods

### Chemicals and materials

E2 (C_18_H_24_O_2_, ≥98%), BPA (C_15_H_16_O_2_, ≥99%), double-stranded salmon testes DNA (%G-C content: 41.2%; molecular mass: 1.3 × 10^6^ Da; DNA fragment lengths: ∼2,000 bp), and *Trametes versicolor* laccase (light brown, ≥0.5 U·mg^−1^) were sourced from Sigma-Aldrich Co., China. Methanol (CH_3_OH, suitable for high-performance liquid chromatography [HPLC], ≥99.9%) and acetonitrile (CH_3_CN, suitable for HPLC, ≥99.9%) were procured from Thermo Fisher Scientific Inc., USA. Tris-HCl buffer solution (10 mM, pH 7), citrate-phosphate buffer solution (CPBS, 10 mM), and Murashige and Skoog (MS) medium were obtained from Aladdin Chemistry Co., China. Stock solutions of E2 and BPA were prepared in methanol at 1 mM. Laccase stock solution (100 U·mL^−1^) and DNA stock solution (dissolving 75.75 nmol of DNA in 100 mL Tris-HCl buffer) were prepared in Milli-Q water. UV absorption at 260 nm with a molar extinction coefficient (*ε*_260_) of 6,600 L·mol^−1^·cm^−1^ was used to determine the DNA concentration in the solution. The DNA purity, indicated by an absorbance ratio at 260 and 280 nm (A_260_/A_280_) of >1.83, confirmed high purity. Wheat seeds (*Triticum aestivum* L.) were supplied by the Anhui Academy of Agriculture Sciences, China. All solutions were prepared using Milli-Q water (18.25 MΩ·cm) and stored at 4 °C prior to use.

### Batch kinetic experiments

The reaction kinetics of EDCs catalyzed by laccase in the presence of DNA were conducted in 20 mL of CPBS at 25 ± 0.5 °C and pH 5 under dark conditions. A concentration of 0.5 U·mL^−1^ laccase was added to solutions containing 5 μM of either E2 or BPA and varying concentrations of DNA (0–7.575 nM). Periodically, 0.5 mL samples were extracted from the reaction mixture, quenched immediately with an equivalent volume of methanol, and filtered through a 0.22-μm membrane for analysis. Experiments were replicated in triplicate, and the oxidation kinetics of EDCs were modeled by pseudo-first-order kinetics:


(1) $$-\frac{d[\mathrm{EDCs}]}{dt}=k_{1}\times [\mathrm{EDCs}],$$

where *k*_1_ is the pseudo-first-order rate constant and [EDCs] is the residual concentration of free EDCs in the reaction system at a given time.

### Enzymatic colorimetric assay

Details on the determination of laccase activity are provided in the Supporting Information.

### Quantification of EDCs and product identification

Quantitative and qualitative analyses, including HPLC and HRMS, are described in the Supporting Information.

### Interaction of DNA with EDCs in laccase catalysis

The interaction mode between DNA and EDCs during enzymatic catalysis was examined using a UV-Vis spectrophotometer (UV-2550, Shimadzu Co., Japan) equipped with 1 cm quartz cuvettes. Samples were analyzed at preset intervals (0, 5, 10, 15, 30, 45, 60, 90, 120, 150, and 180 min) for UV absorption spectrum (220–300 nm) in 1-nm increments at 25 ± 0.5 °C. ATR-FTIR spectroscopy (NEXUS-870, Thermo Nicolet Co., USA) and CD spectroscopy (Chirascan, APL, UK) were also employed to further explore the binding mechanism between DNA and EDCs.

The morphological changes of DNA binding to EDCs and their oligomers were directly observed by AFM. DNA (7.575 nM) and EDCs (5 μM) were incubated at 25 ± 0.5 ° C and pH 5.0 to ensure that EDCs were adequately bound to DNA. Next, the enzymatic reaction was initiated by adding 0.5 U·mL^−1^ laccase to the mixture. By adjusting the pH of the mixture to 1.5, the enzymatic reaction was periodically terminated. To prepare the sample for AFM, 50 μL of the mixed solution was pipetted onto freshly cracked mica sheets, gently washed with ultrapure water and air-dried, and then imaged by AFM (Dimension Icon, Bruker Co., USA). AFM images were recorded at a resolution of 500 × 500 pixels and a scanning line speed of 0.600–0.998 Hz.

### Microscopic and spectroscopic analyses of precipitated polymers

Precipitated polymers collected from the laccase-induced polymerization transfer of EDCs were freeze-dried, ground, and sieved. Their microstructures, functional groups, and chemical compositions were characterized using SEM (Regulus 8100, Hitachi, Japan) equipped with an EDS (Bruker Co., Germany), FTIR, and ^13^C-NMR (Avance III, Bruker Co., Germany).

### Molecular dynamics simulation

MDS was performed to model the interaction of DNA with EDCs and their oligomers (dimer, trimer, and tetramer). The 3D structure of B-DNA dodecamer d(CGCGAATTCGCG)_2_ was sourced from the Protein Data Bank (PDB ID: 2B0 K). The structures of EDCs and their oligomers were built using ChemBioDraw Ultra 14.0, optimized with ChemBio3D Ultra 14.0, with nonpolar hydrogen atoms merged and Kollman charges added using MGLTools 1.5.6. Simulations were conducted using GROMACS 2016.4 with the AMBER99SB force field. Rmsd was calculated to assess the stability of each complex:


(2) $$\mathrm{rmsd}=\sqrt{\frac{1}{N_{\mathrm{atm}}}\sum_{A=\;1}^{N_{\mathrm{atm}}}\;{\left(r_{A}-r_{A}^{\mathrm{ref}}\right)}^{2}},$$

where $N_{\mathrm{atm}}$ is the number of atoms, $r_{A}$ is the position of atom *A*, and $r_{A}^{\mathrm{ref}}$ is the reference position of atom *A*.

### Phytotoxicity assay

The phytotoxicity of DNA and EDC solutions treated by laccase-induced polymerization was evaluated using a GI. Wheat seeds were monitored over a 3-day cultivation period in MS medium under controlled conditions. The GI was calculated using the formula:


(3) $$\mathrm{GI}\;(\text{\%})=\frac{N_{\mathrm{CRS}}\times R_{\mathrm{CRS}}}{N_{\mathrm{MW}}\times R_{\mathrm{MW}}}\times 100,$$

where *N*_CRS_ and *N*_MW_ are the number of seeds germinated in the reaction solution and Milli-Q water, respectively, and *R*_CRS_ and *R*_MW_ are the root lengths in the reaction solution and Milli-Q water, respectively.

## Supplementary Material

pgaf148_Supplementary_Data

## Data Availability

All data are included in the article and Supporting Information.
