# Supplementary material for: Molecular insights into the interaction mechanism of endocrine-disrupting chemicals and DNA in laccase-induced polymerization transfer
Source: PNAS Nexus. 2025 May 12;4(5):pgaf148. doi: 10.1093/pnasnexus/pgaf148 (PMC12082286; doi:10.1093/pnasnexus/pgaf148)
Supplement: pgaf148_Supplementary_Data [file pgaf148_supplementary_data.docx]

***Supporting Information***

**Molecular insights into the interaction mechanism of EDCs and DNA in laccase-induced polymerization transfer**

Kai Sun^a,b^, Zeyu Shi^a^, Lingzhi Dai^a^, Youbin Si^a^, Junchao Ma^c^, Hui Lin^d^, Han-Qing Yu^b,*^

^a^Institute of Ecological Environmental Protection and Pollution Remediation Engineering, College of Resources and Environment, Anhui Agricultural University, Hefei, 230036, China

^b^CAS Key Laboratory of Urban Pollutant Conversion, Department of Environmental Science and Engineering, University of Science and Technology of China, Hefei, 230026, China

^c^College of Resources and Environmental Sciences, Nanjing Agricultural University, Nanjing, 210095, China

^d^Research Center for Eco-Environmental Engineering, Dongguan University of Technology, Dongguan, 523808, China

**This PDF file includes:**

Supplementary text

Figures S1 to S4

Tables S1

**Text S1**

**Determination of laccase activity**

2,6-dimethoxyphenol (DMP) is generally used as the chromogenic substrate to evaluate the catalytic activity of laccase. For evaluating enzyme activity, 3.4 mL 10.0 mM CPBS (pH 3.8) containing 1.0 mM DMP contacted with 20 μL reaction solution was immediately incubated at 25±0.5 °C for 5 mins under static conditions. The DMP oxidation products were quantitatively analyzed by recording an absorbance of 468 nm (UV-Vis spectrophotometer, UV-2550, Shimadzu Co., Japan). A unit of enzyme activity (U·mL^-1^) is defined as the amount of laccase that elicited a unit change per minute in absorbance at 468 nm. The relative activity of laccase in the reaction system was calculated using the formula:

$Relative activity \left( \% \right)=\frac{{LA}_{t}}{LA_{0}}\times100\%$ (1)

where *LA*_0_ is the initial laccase activity and *LA*_t_ is the laccase activity in the reaction system at a given time.

**Text S2**

**Quantitative and qualitative analysis**

The concentrations of EDCs (E2 and BPA) in the reaction solutions were quantified by an HPLC system (Waters Co., USA) equipped with a 600 pump, a 2707 autosampler, and a 2998 photodiode array detector. Chromatographic separation was achieved using an ZORBAX Eclipse Plus C18 column (4.6 mm × 150 mm, 5 μm particle size, Agilent Co., USA) maintained at 40 °C. The mobile phase consisting of acetonitrile and water (70: 30, v: v) was pumped through the column at a flow rate of 1.0 mL·min^-1^. The detection wavelength was set at 280 nm, which was the maximum absorption wavelength observed for E2 by UV-Vis spectrum. 20 µL sample was introduced onto the HPLC system every 15 mins. E2 and BPA concentrations were quantified from the chromatogram by peak area based on the multipoint standard calibration curves.

Possible oxidation products of EDCs induced by laccase catalysis were recognized using a Triple TOF™ 5600+ high-resolution mass spectrometer (HRMS; AB SCIEX Co., USA). The reaction solution was extracted with ethyl acetate thrice and concentrated to near dryness with the same methods. The mass spectrometer was operated in negative electrospray ionization (ESI) mode. The ESI source operated using N_2_ as the drying/carrier gas. The operation parameters of the negative ion mode were as follows: Capillary temperature, 350 °C; capillary voltage, −30 V; electrospray voltage, 4.5 kV; tube lens voltage, −110 V. The sheath gas (N_2_) and the auxiliary gas (He) had a flow rate of 80 and 30 arbitrary units, respectively. Total ionization chromatography was collected in a mass scan range of *m*/*z* 100-1500 to identify the intermediate products of EDCs with high precision (mass error < 5 ppm). Data analysis was acquired through Thermo Scientific™ Xcalibur™ Software, version 3.0, USA.


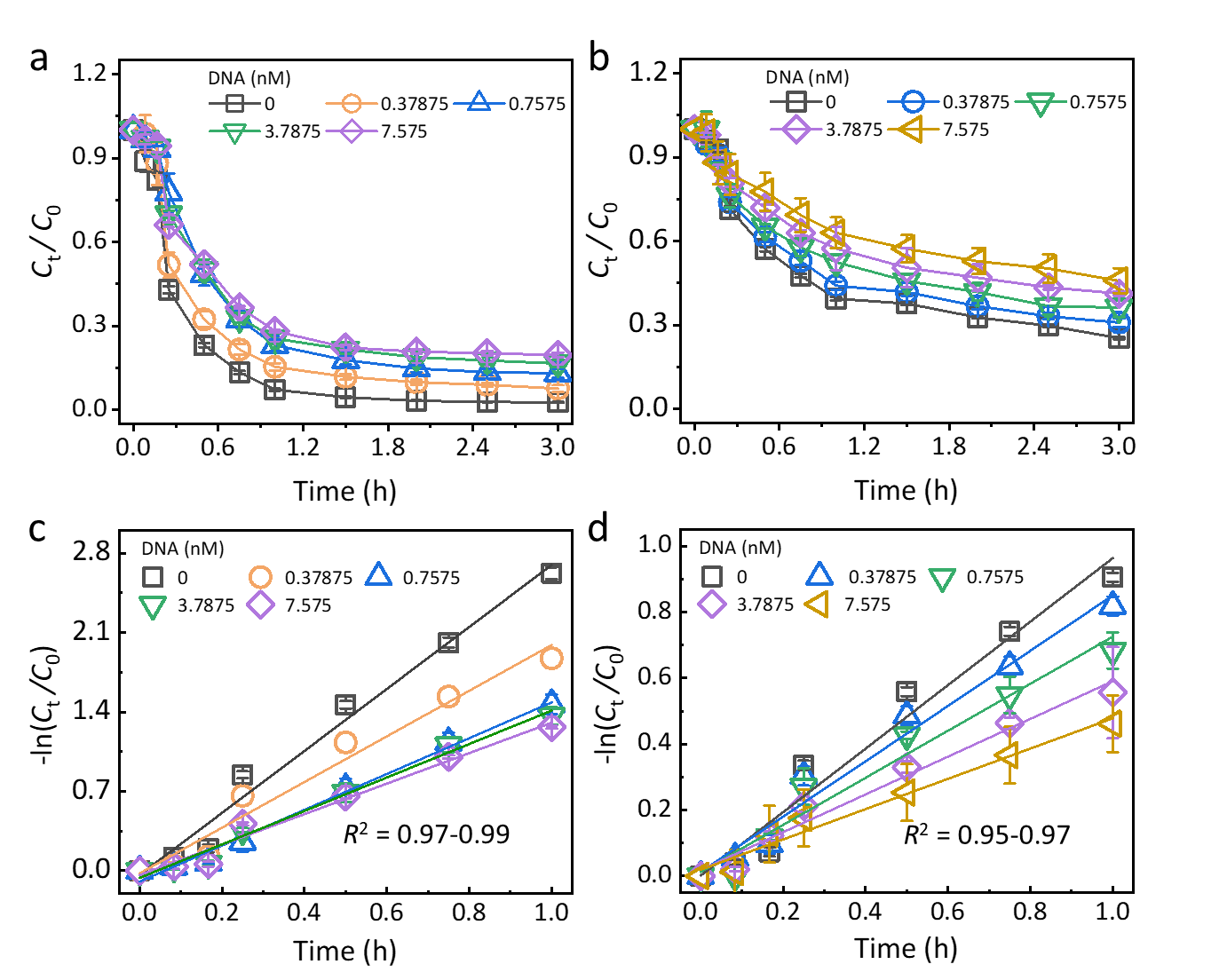


**Figure S1.** Effect of DNA concentration on EDC oxidation by laccase catalysis. (a) E2 oxidation; (b) BPA oxidation; (c) Pseudo-first-order kinetic model for E2 oxidation within 1 h; (d) Pseudo-first-order kinetic model for BPA oxidation within 1 h.

**
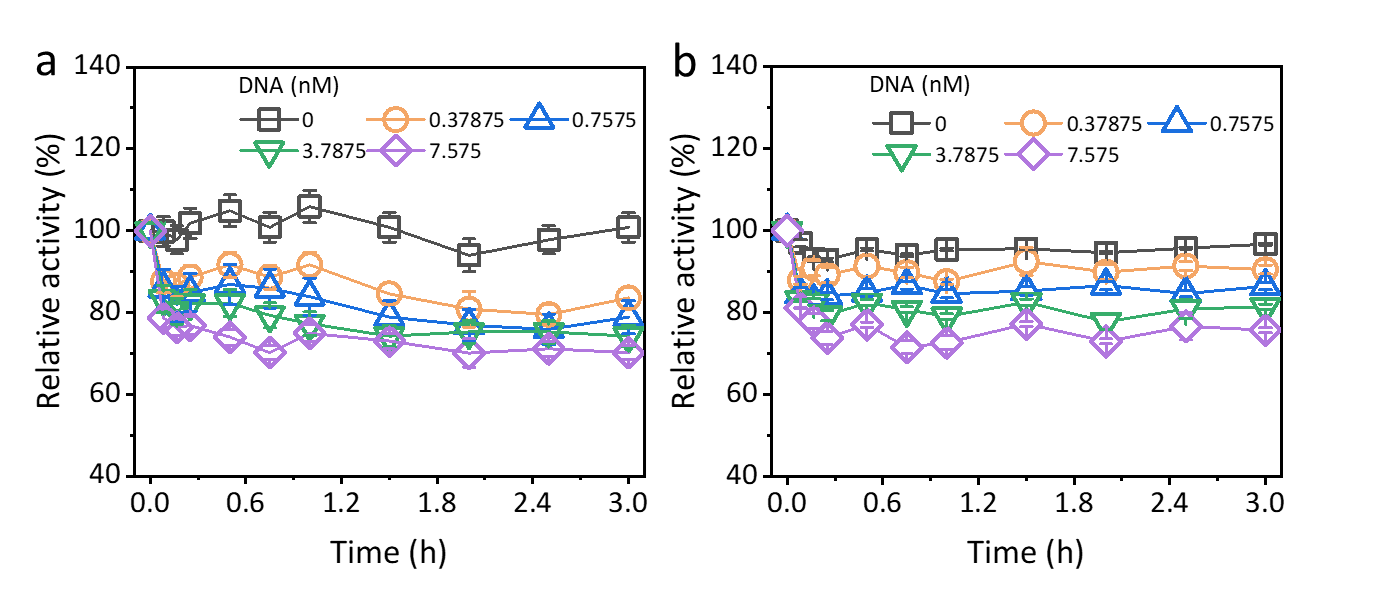
**

**Figure S2.** Effect of DNA concentration on laccase activity during the enzyme-induced oxidation of EDCs. (a) E2; (b) BPA.


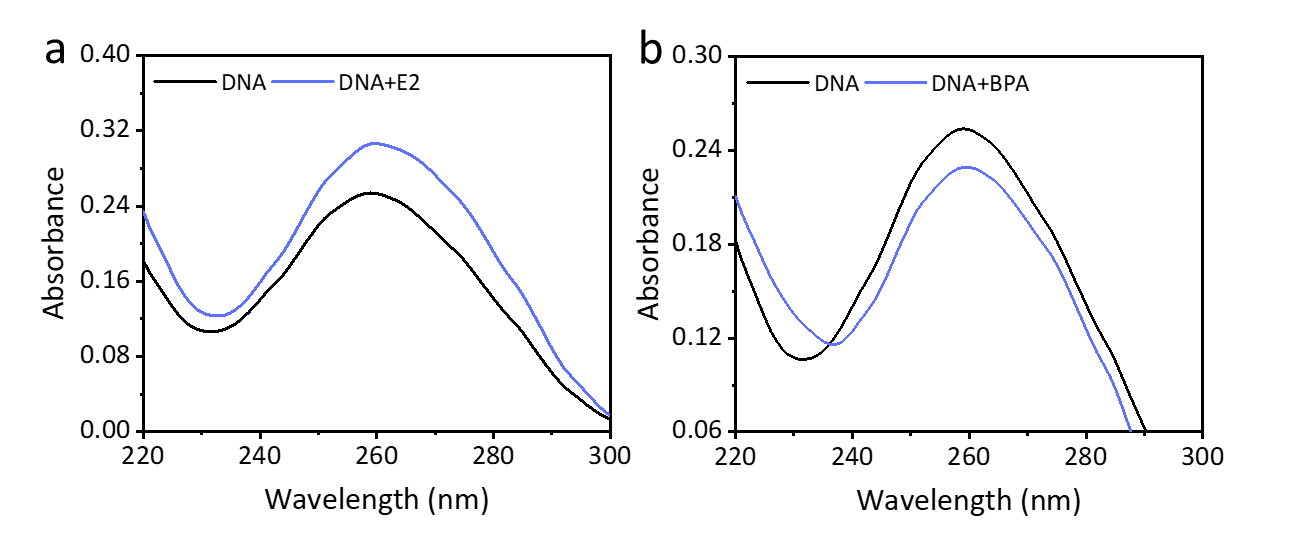


**Figure S3.** The UV-Vis absorption spectra of DNA and DNA-EDC systems. (a) DNA-E2; (b) DNA-BPA.

**
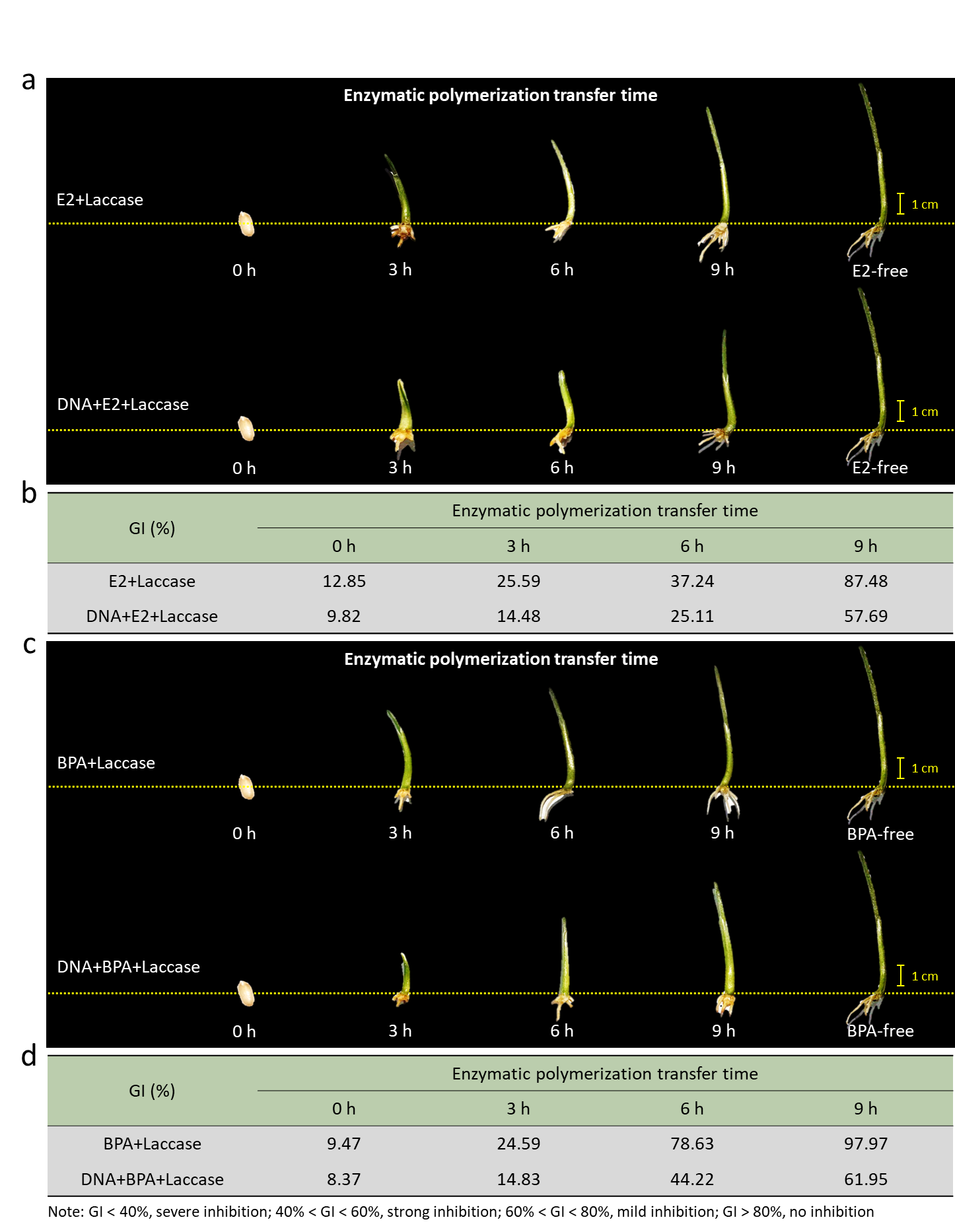
**

**Figure S4.** Phytotoxicity and germination index (GI) of DNA and EDC solutions treated by laccase-induced polymerization on wheat seeds. (a) Observations of growth changes in wheat seeds treated with DNA and E2 solutions post-laccase treatment. (b) Germination Index (GI) of wheat seeds during laccase-induced polymerization of DNA and E2 solutions. (c) Growth alterations in wheat seeds treated with DNA and BPA solutions post-laccase application. (d) Germination Index (GI) for wheat seeds during laccase-induced polymerization of DNA and BPA solutions.

**Table S1.** The theoretical and experimental *m*/*z* values for the intermediate products of EDCs in laccase-induced EDC-DNA systems

| EDCs | Molecular formula | *m/z* ([M-H]^-^) | | Mass error (ppm) | Chemical structure |
| --- | --- | --- | --- | --- | --- |
|  |  | Experimental value | Theoretical value |  |  |
| E2 | C_18_H_24_O_2_ | 271.1693 | 271.1698 | 1.8 | E2 |
|  | C_18_H_22_O_2_ | 269.1548 | 269.1541 | –2.4 | E1 |
|  | C_36_H_46_O_4_ | 541.3310 | 541.3318 | 1.4 | E2 dimer |
|  | C_54_H_68_O_6_ | 811.4930 | 811.4937 | 0.9 | E2 trimer |
|  | C_72_H_90_O_8_ | 1081.6555 | 1081.6557 | 0.2 | E2 tetramer |
| BPA | C_15_H_16_O_2_ | 227.1074 | 227.1072 | –0.9 | BPA |
|  | C_6_H_6_O | 93.0339 | 93.0340 | 1.5 | Phenol |
|  | C_9_H_11_O | 134.0735 | 134.0732 | –2.5 | Dimethylphenol |
|  | C_30_H_30_O_4_ | 453.2071 | 453.2066 | –1.2 | BPA dimer |
|  | C_45_H_44_O_6_ | 679.3054 | 679.3059 | 0.8 | BPA trimer |
|  | C_60_H_58_O_8_ | 905.4067 | 905.4053 | –1.5 | BPA tetramer |
